# Development of a human analogue ADHD diagnostic system for family dogs

**DOI:** 10.1038/s41598-025-09988-8

**Published:** 2025-07-16

**Authors:** Barbara Csibra, Nóra Bunford, Márta Gácsi

**Affiliations:** 1 Institute of Biology, Department of Ethology, Eötvös Loránd University, Pázmány Péter sétány 1/C, Budapest, 1117 Hungary; 2 Clinical and Developmental Neuropsychology Research Group, Research Centre for Natural Sciences, Institute of Cognitive Neuroscience and Psychology, Magyar tudósok körútja 2, Budapest, 1117 Hungary; 3 HUN-REN-ELTE Comparative Ethology Research Group, Pázmány Péter sétány 1/C, Budapest, 1117 Hungary

**Keywords:** ADHD, Inattention, Hyperactivity, Impulsivity, Questionnaire, Functional impairment, Animal model, Family dog, Animal behaviour, Human behaviour

## Abstract

Dogs exhibit natural variability in inattention, hyperactivity and impulsivity traits, sometimes with extreme manifestations resembling to Attention-Deficit/Hyperactivity Disorder (ADHD) symptoms in humans. Using human-standard diagnostic methods, we developed a comprehensive approach for supporting diagnosing ADHD in dogs (*N* = 1872) based on a validated questionnaire that consists of two parts: a symptom section that provides factor scores for three areas (inattention, hyperactivity and impulsivity), and a functionality section that evaluates the extent to which these symptoms cause functional impairments. Based on the functionality section, we classified dogs as functionally impaired if they showed impairment in at least 4 out of the 7 questions of at least one area (*n* = 116; 6.2% of the sample). Then, we projected the impaired–non-impaired categories on the ADHD total scores of the symptom section to establish potential cutoff values. The ROC curve analysis resulted in an excellent AUC value (0.861). A cutoff score of 26 was established based on well-defined sensitivity and specificity trade-offs. Finally, we applied the combined thresholds of the two parts of the questionnaire to identify dogs at-risk vs. non-at-risk for ADHD (*n* = 79; 4.22% of the sample). We offer the first replicable method to screen dogs with suspected ADHD. A final diagnosis could be set after dog-owner pairs participate in relevant behavioural tests and an expert consultation. Allowing for comparisons between ADHD vs. typical groups of family dogs, our method not only facilitates the improvement of the wellbeing of at-risk dogs, but also makes it more feasible to use dogs as a natural model species for studying ADHD.

**Supplementary Information:**

The online version contains supplementary material available at 10.1038/s41598-025-09988-8.

## Introduction

Attention-Deficit/Hyperactivity Disorder (ADHD) is among the most common human neurodevelopmental disorders, with childhood and adolescent global prevalence estimates around 5–9%^1–3^. Among adults, the prevalence is estimated to be lower, ranging between 2 and 4%^4^. There are more and more children diagnosed with ADHD, but there is still a debate whether it is a result of more sophisticated screening tools, differently applied diagnostic criteria, or ADHD is simply occurring more and more often^4–6^. Beyond inattention, hyperactivity and impulsivity symptoms, individuals with ADHD exhibit functional impairments, including the academic and social domains^7^. In humans, questionnaires combined with clinical interviews and behavioural assessments are efficient tools to assess ADHD symptoms and form the basis of diagnosis.

Ample evidence indicates that family dogs could serve as a potential animal model of ADHD, as dogs naturally display phenotypic variability regarding attention, activity, and impulsivity^8–13^including extreme manifestations of these traits^12^. Moreover, pet dogs have been reported to show functional impairments^14,15^ demographic risk factors and related comorbidities^12,16,17^ comparable to humans. In dogs, similar to what was observed in human ADHD, the severity of functional impairments correlated with the degree of ADHD-like symptoms^14^. These similarities in displayed behaviours to human ADHD suggest a functional overlap between dogs and humans, but it is still uncertain to what extent these traits are similar to human ADHD. In veterinary practice, an increasing number of dogs are being diagnosed with behaviours resembling to human ADHD, mainly referred to as ‘Hypersensitivity–Hyperactivity Syndrome’ (HSHA)^18–20^. Although emerging evidence indicates that a condition similar to human ADHD exists in dogs, the diagnosis is even debated among veterinarians due to the absence of uniform definitions, objective measurement tools, and standardized diagnostic criteria^14,16,21^. These uncertainties and subjectivity in diagnosis complicate the replication of findings in the field of ‘canine ADHD,’ and that is why researchers often refer to these dogs as having ‘ADHD-like’ symptoms^14,21^.

Diagnosing ADHD in children depends on a set of strict criteria. According to the Diagnostic and Statistical Manual of Mental Disorders (DSM-5-TR)^22^) the following criteria must be met to establish the diagnosis of ADHD: a persistent pattern of inattention and/or hyperactivity-impulsivity symptoms which interfere with functioning or development, as characterized by inattentive or hyperactive-impulsive symptoms to a degree that is inconsistent with developmental level and that negatively impacts directly on social and academic/occupational activities. Regarding symptomatology in detail, the diagnostic criteria for individuals under the age of 17 years require 6 or more symptoms of inattention and/or 6 or more symptoms of hyperactivity and impulsiveness out of 9 symptoms of each dimension (the symptoms display continuously for at least 6 months and started to show the symptoms before the age of 12). For individuals aged 17 years or older, only 5 symptoms are required to be present to meet the diagnostic criterion. Furthermore, to establish the diagnosis of ADHD, the DSM requires evidence of symptomatology in more than one setting (e.g., at home, school), and there must be clear evidence that the symptoms interfere with, or reduce the quality of, social, academic, or occupational functioning. Another diagnostic criterion is that the symptoms are not better explained by another mental disorder (e.g., mood disorder, anxiety disorder, dissociative disorder, personality disorder, substance intoxication, or withdrawal) (DSM-5-TR)^22^).

According to current guidelines, the clinical diagnosis of suspected ADHD should include a comprehensive review of prenatal, perinatal, medical, and family history, academic performance, environmental factors, and a detailed physical examination^23^. A mental health evaluation should be undertaken to investigate the presence of comorbid illnesses and identify significant impairment. Behaviour rating scales completed by parents and teachers are primarily used to assess ADHD symptoms and related impairments, as children cannot accurately evaluate their own symptoms^24–27^. Although the DSM-5 provides specific diagnostic criteria, the actual diagnosis often depends on subjective evaluation of parents’ reports rather than objective measures^28,29^. The main reliance on parent and teacher reported symptoms raises concerns about the reliability and subjectivity of ADHD diagnosis^30^ highlighting the complexity and potential lack of objectivity in the diagnostic process. The difficulty of diagnosing and defining human ADHD is well reflected by the fact that the diagnostic criteria have dynamically changed and evolved through various DSM publications over the decades^23^and in parallel with newer research findings in the topic, there is a continuous debate regarding the exact criteria of ADHD diagnosis^31,32^.

In dogs, currently used questionnaires assessing ADHD-related characteristics^13,33^ are not suitable to detect diagnosable individuals with ADHD^14,34^. The Dog ADHD Rating Scale (Dog ARS)^13^), the most extensively used rating scale for evaluating ADHD-like behaviours in dogs^8,9,11,12,20,35,36^ does not include functionality assessment, which would be essential to determine diagnostic criteria (see Csibra et al., 2024a, 2022). Of note, studies investigating ADHD-like traits in dogs have predominantly used ADHD questionnaire scores as continuous variables, conducted on convenience dog samples that were not specifically selected or grouped based on extreme ADHD-like behaviour^14^. This approach contrasts with typical human ADHD research, which mostly involves comparing distinct ADHD and non-ADHD groups. Only one study to date has employed the Dog ARS^13,37^ to evaluate its applicability as a diagnostic tool on HSHA syndrome^20^. In this study, the Dog ARS was applied to a group of dogs already diagnosed with HSHA by veterinarians and to a matched control group. However, the input for the ROC analysis was predetermined based on the veterinarians’ assessments of diagnosed HSHA group versus normal group, relying on a limited set of criteria that may not necessarily be ADHD-specific (e.g., lack of or delay in acquiring bite control in a puppy over 2 months of age). Furthermore, the study did not detail the exact, replicable methods used to assess HSHA symptoms, such as the specific questions asked by veterinarians or how owners’ responses were used to categorize dogs into HSHA versus normal groups. The authors themselves highlighted limitations, such as including inconsistent application of diagnostic criteria due to the involvement of large number of veterinarians, and potential bias in selection of the control group, such as choosing very calm dogs, all within a context where there is no standardized ADHD definition for dogs^20^.

Recently, a validated ADHD questionnaire for measuring family dogs’ inattention, hyperactivity, impulsivity, and also functionality was developed (Dog ADHD and Functionality Rating Scale, hereafter: DAFRS^14^).

However, the DAFRS does not yet distinguish between typical and pathological levels of impulsivity, hyperactivity, and inattention, even though recent behavioral studies suggest that dogs exhibit problematic behaviors similar to ADHD (e.g^38,39^.), which, according to both the owners and the veterinarians, manifest themselves in functional deficiencies that require intervention^14,18,19,21,40^. Currently, the assessment of these conditions in practice is challenging due to the lack of standardized measurement tools. Thus, it is crucial to develop a sound methodology to help practical screening of dogs suspected of having ADHD.

## Aims

Our aim was to determine whether family dogs can be categorized as being at-risk for ADHD based on their DAFRS total scores (number of symptoms) and the degree of their functional impairments, and develop a replicable tool as objective as possible to identify such individuals. To evaluate the applicability of DAFRS as a diagnostic tool, we employed standard diagnostic methods and criteria applied in human diagnostic assessments, where both the presence of symptoms and the severity of functional impairments are considered for diagnosis^22,41^. Of note, while the differences between the typical lives of humans (children) and dogs prevented a complete adaptation of human assessment methods, we aimed to use comparable standard approaches and the most objective, replicable methods possible.

## Methods

### Ethics statement

All procedures were carried out in accordance with relevant guidelines and regulations for human participants (dog owners) as volunteers participating in the study. Prior to participation, owners received detailed information about the aims, circumstances, and features of the study. The study was carried out in accordance with the Declaration of Helsinki and approved by the Hungarian Ethics Committee of “United Ethical Review Committee for Research in Psychology (EPKEB)” (reference number of approval: EPKEB-2023-04). Owners gave informed consent to participate in the online questionnaire study (see owner consent form and privacy policy information in Supplementary material, Appendix A).

### Subjects

Participants were recruited through the Department of Ethology (Eötvös Loránd University in Budapest, Hungary) participant pool and website, popular social networking sites, and via snowball sampling. We included previously obtained DAFRS questionnaire data from Csibra et al.^14^ on *n* = 1168 dogs and collected additional data from applicants to achieve a final sample size of *N* = 1872 for our analyses. Only dogs older than 10 months were included in the sample, as behaviour undergoes significant changes up to 10 months due to maturation^42^further, based on sleep electrophysiology parameters, dogs’ central nervous system is not fully mature until 10 months of age^43^. Thus, the sample included dogs aged 10 to 237 months (*M*age = 56.19 months, *SD* = 37.43), from 142 distinct breeds as well as 513 mixed breed dogs. The sample consisted of 909 male and 963 female dogs, 1255 intact and 617 neutered dogs.

## Measures

### Rating scale measures

Individual differences in dogs’ inattention, hyperactivity, and impulsivity were measured using the DAFRS^14^; a 17-item (6 items measuring inattention, 4 items measuring hyperactivity, and 7 items measuring impulsivity) owner-reported questionnaire tool (see Appendix A for the questionnaire). Owners indicate the frequency with which their dog behaves as described in each item (ranging from “never” = 0 to “very often” = 3). The ADHD total score is calculated as the sum of the responses to these questions, with a maximum achievable ADHD total score of 51 points (17*3). DAFRS was developed in collaboration with a clinical expert and researcher who had extensive experience in ADHD and associated problems in humans, further, the entire development process was evaluated and discussed with veterinarians specializing in dog behaviour, including a diplomat from ECAWBM and a European Veterinary Specialist in Behavioral Medicine. The development also involved contributions from ethologists, clinicians and researchers specializing in human ADHD, and certified dog trainers. The internal consistency, test–retest reliability, interrater reliability, and convergent validity of the DAFRS were evaluated in our previous study^14^. According to our previous findings, the DAFRS subscales showed good (inattention and hyperactivity) and excellent (impulsivity) internal consistency. Further, test–retest analyses demonstrated excellent agreement between measurements for all subscales. Interrater reliability analyses showed fair (inattention) to moderate (hyperactivity and impulsivity) agreement between dog trainers and owners, consistent with on human teacher-parent rating comparison data. The DAFRS also demonstrated convergent validity with prior studies on dogs in terms of associations with age, sex, neutering and training status and ADHD factor scores^12,13,37^. In our current analyses, we applied the factor structure established in our earlier study^14^ but the aforementioned psychometric analyses were not re-run on the present dataset.

We treated the ADHD subscale questionnaire responses—serving as indicators of symptom severity—as scores, employing the continuous scoring method^44^. This approach differs from the traditional method used in human diagnostics, which typically relies on the number of symptoms present^44–46^. We used scores for practical reasons, as it is simpler for users than converting responses to each item into binary presence or absence of symptoms for each evaluation.

Due to the varying number of items per ADHD subscale, we applied a weighting procedure to ensure that each subscale contributes equally to the ADHD total score. Appendix B provides a detailed description of the weighting calculation process (see Supplement). The weighted ADHD scale scores (range: 0 to 63) derived from this process were used in the further analyses.

#### Functionality measurement

The latest ADHD rating scales for humans classify functional impairment questions into six impairment domains: relationships with significant others, peer relationships, academic functioning, behavioural functioning, homework performance, and self-esteem (ADHD-RS-V^45,47^). However, many of the functional impairment questions used in human assessments are challenging to apply to dogs (e.g., not all dogs attend classes in dog schools). Therefore, we chose to categorize functionality-related items connected to the three ADHD factors by applying questions that are specifically relevant to dogs and cover different contexts of their lives.

The DAFRS consists of questions about dogs’ functioning related to inattention, hyperactivity, impulsivity, and aggression. Of note, from this analysis we excluded the aggression-related items, as in dogs these behaviours can be strongly influenced by factors beyond ADHD traits (e.g., gender, neutering status, breed, training and environmental stressors); see Supplement, Appendix A for details on omitted items.

For each of the three areas (functionality-inattention, functionality-hyperactivity, functionality-impulsivity), there are 7 similarly worded questions varied by the relevant ADHD characteristic, where the 0–3 scale indicates the extent to which the behaviour problems (if they occur) were attributable to the dog’s inattention/hyperactivity/impulsivity. For example, for impulsivity functional problem category, the ratings were as follows: (0) There is no such problem/It is a problem, but not a result of impulsivity; (1) It is a problem, and, to some extent, is a result of impulsivity; (2) It is a problem, and, more or less, is a result of impulsivity; (3) It is a problem, and is largely a result of impulsivity (Supplement, Appendix A).

## Data analysis

Following practices in human diagnostics, we aimed to establish criteria that integrate the ADHD total score and the degree of functional impairment (ADHD-RS-5)^45,47^). The analysis consisted of three main steps.

First, dogs were considered impaired if they received a rating of 2 or 3 (on a 0–3 scale) in at least 4 out of the 7 questions in at least one of the three ADHD-related functional areas (see Appendix A). We based our criteria on a common practice in human rating scale measurements for identifying the affected group and separate from developmentally normative behaviour^47^. The result of this categorization (impaired vs. non-impaired) was used in the Receiver Operating Characteristic (ROC) analysis as the input variable to assess the diagnostic accuracy of the DAFRS questionnaire. ROC analysis is a statistical method used to evaluate the accuracy of diagnostic tests by analysing their ability to distinguish between disorder presence and absence, based on sensitivity and specificity across varying cutoff values^48,49^. The analysis uses a state variable (impaired vs. non-impaired) and a test variable (weighted and rounded ADHD total scores). For more details on ROC analysis, see Supplement, Appendix C.

While it is generally considered more important in screening tools to maximize sensitivity to avoid missing positive cases –even at the cost of increasing false positives, acknowledging that the determination of the cutoff is somewhat arbitrary also in human measures^50,51^ in the case of dogs we prioritized specificity over sensitivity. As our method represents a preliminary step towards more objective identification of dogs at-risk for ADHD, we argue that the risk of identifying more false positives may result in unnecessary interventions, such as potential medication of young dogs^18^. This emphasizes the necessity of prioritizing specificity over sensitivity, to minimize the risk of misdiagnosis and focus resources on those dogs which require further evaluation and intervention.

Finally, we applied combined thresholds of the functional impairment classification (impaired vs. not-impaired) and the determined cutoff of the ADHD total scores to identify individuals as at-risk vs. non-at-risk for ADHD.

Statistical analyses were conducted in IBM SPSS Statistics version 27.0.1.0.

## Result

**Fig. 1:**
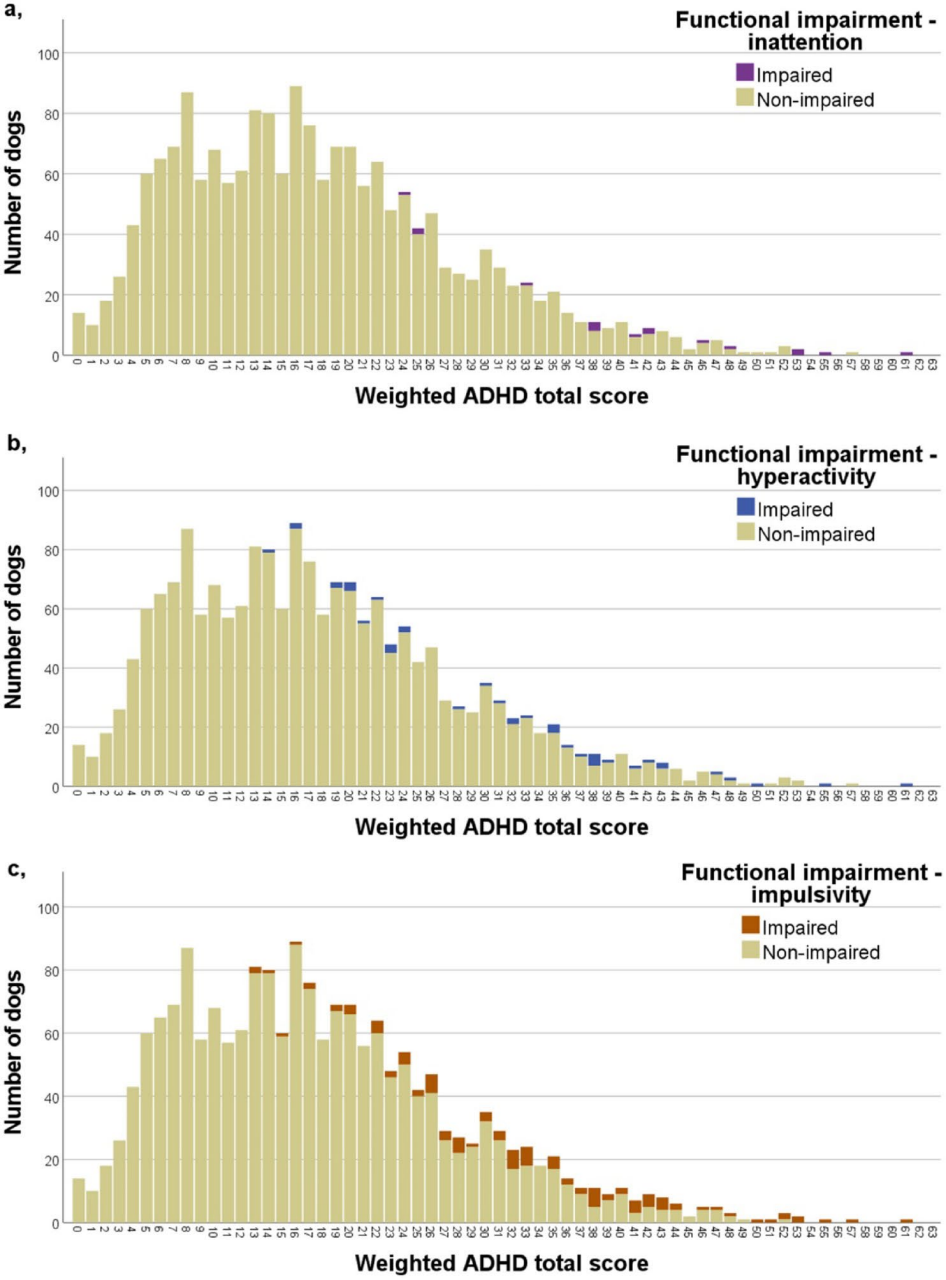
Number of functionally impaired dogs sorted by their ADHD total score (with a rating of ≥ 2 in the case of ≥ 4 questions) in the inattention **(a)**, hyperactivity **(b)** impulsivity **(c) **areas. (None of the dogs received a weighted ADHD total score of 54, 56, 58, 59, 60, 62, 63 in the sample.).

As a first step, we determined the number of dogs meeting the impaired criteria (have a rating of ≥ 2 in ≥ 4 questions in at least one of the three ADHD-related functionality areas: functionality-inattention, functionality-hyperactivity, functionality-impulsivity). These results are presented in Fig. 1. (a), (b), (c), showing the proportion of functionally impaired dogs separately for each functionality areas, sorted by their ADHD total score.

Summarizing the results of the three impairment areas, a total of 116 dogs were classified as functionally impaired (6,2% of the sample), meeting the criteria for at least one area. With respect to the functional impairment areas, 16 dogs met the criteria for inattention, 40 for hyperactivity, and 101 for impulsivity. Regarding the combinations of the impairment areas, 8 dogs met the criteria for both inattention and hyperactivity, 13 for inattention and impulsivity, 28 for hyperactivity and impulsivity, and 8 for all three areas.

Secondly, impaired vs. non-impaired classification was used as the input variable for the ROC analysis to test the effectiveness of the questionnaire in distinguishing dogs with at-risk for ADHD. The AUC indicated that the test has an excellent discriminatory ability (AUC = 0.861; 95% CIs [0.832; 0.890], consistent with standards for diagnostic tests^52^.

**Fig. 2:**
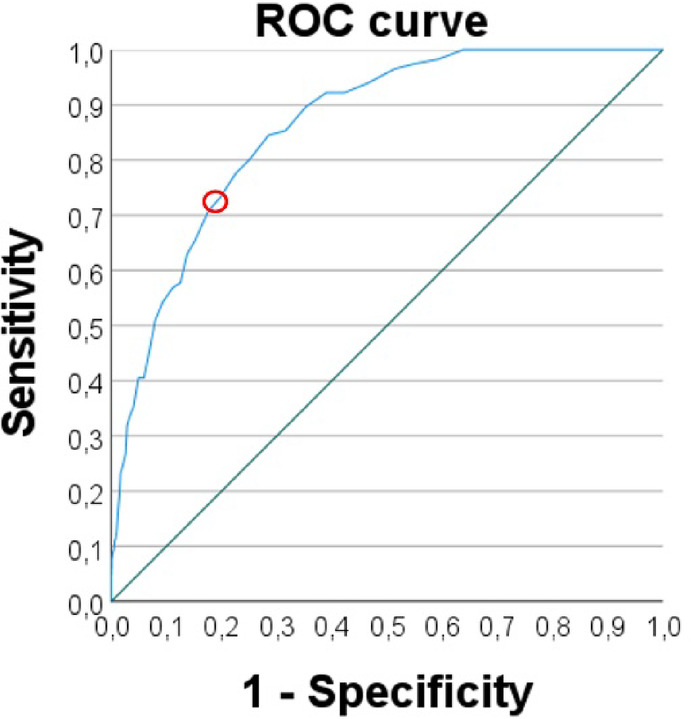
The ROC curve for weighted ADHD total scores for 1872 dogs. The red circle mark represents the selected cutoff point; sensitivity = 71 specificity = 82.

When deciding on the cutoff point on the weighted ADHD total scores, we considered the features of the ROC curve, and the trade-off of the sensitivity and specificity values. The sensitivity and specificity values for various weighted ADHD total score thresholds are presented in the Supplement, Appendix D. Based on our objectives to prioritise specificity (see methods), we determined the sensitivity = 71 and specificity = 82 cutoff point. These values are considered good and excellent respectively, according to established criteria^53,54^. The sensitivity and specificity values collectively determined a score value of 25.5 (rounded to 26) for the weighted ADHD total score as cutoff point for at-risk ADHD, that is, dogs with score 26 or more were categorized as at-risk individuals. Hereafter, these rounded scores will be provided as the cutoff values used for further analysis (Fig. 2).

**Fig. 3:**
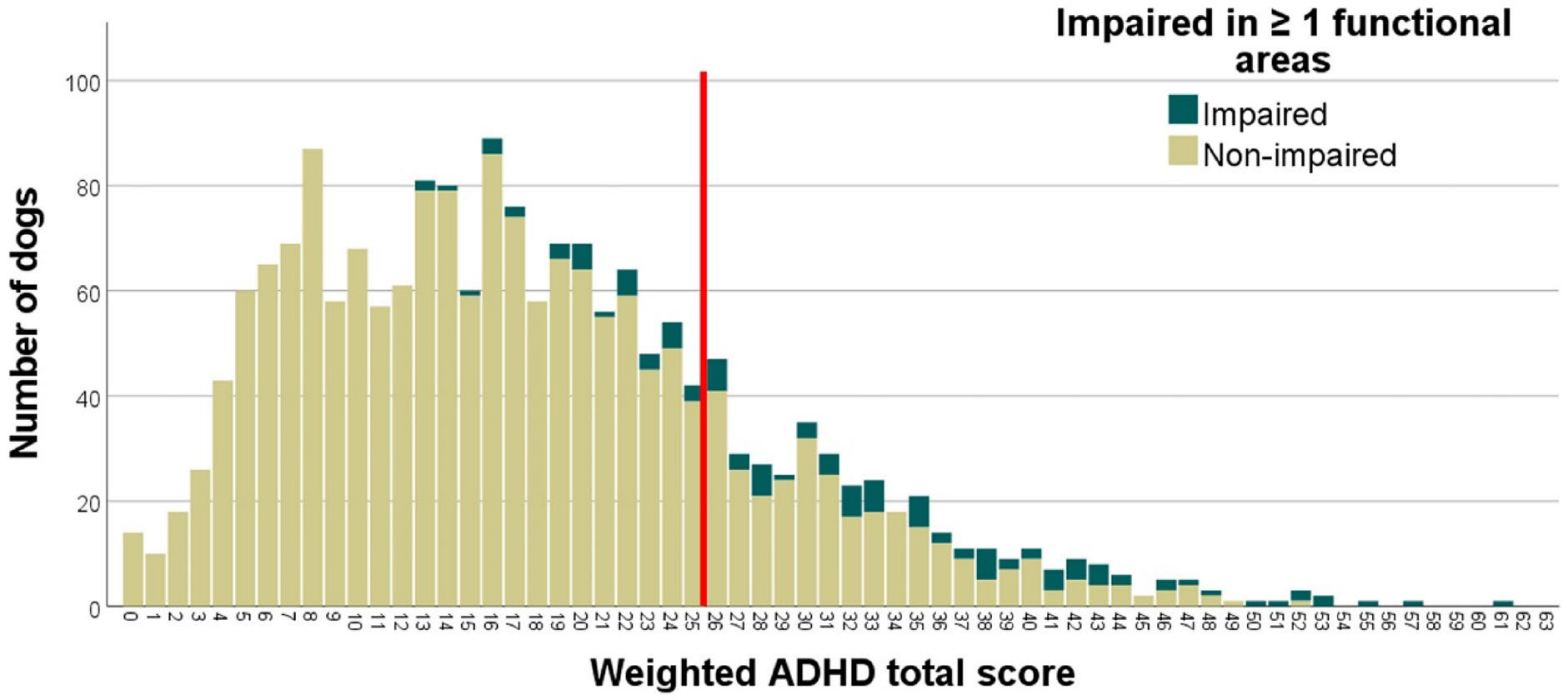
Distributions of the weighted ADHD total scores (total height of columns) and functional impairment (green marker). The red line indicates the cutoff point on the weighted ADHD total scores. Exceeding the cutoff value (≥ 26) combined with the presence of impairment in at least one functional area (green marker) together determine dogs at-risk for ADHD.

Considering only the ADHD weighted total score cutoff without the functional impairment criteria, *n* = 362 dogs scored above the cutoff point (19,34% of the sample; see all dogs to the right of the red marker on Fig. 3.).

Third, as the diagnosis of human ADHD is based on the presence of both functional impairments and ADHD symptoms, a combination of functional impairment scores and the cutoff of the ADHD weighted score was applied to determine the final diagnostic criteria. That is, dogs had to have a rating of ≥ 2 in ≥ 4 questions in at least one functional area and an ADHD total score of ≥ 26. Based on this calculation, finally 79 dogs were classified as at-risk for ADHD (4.22% of the sample; see Fig. 3.). Detailed information on sample demographics of the at-risk dogs (breed, age, sex, neutering status) are presented in the Supplement, Appendix E.

## Discussion

The aim of this study was to develop a method for identifying family dogs at-risk for ADHD, using a diagnostic approach analogous to those used in human ADHD research. The developed method constitutes an essential first step toward establishing a human analogue diagnostic framework based on replicable methodology.

It should be noted that, unlike in human studies, where the effectiveness of diagnostic tools are typically tested against clinically validated diagnoses (forming the basis for ADHD and non-ADHD groups for ROC input^51,55^the present ROC analysis for dogs relies solely on owner-reported questionnaire data. This difference is due to the lack of a detailed, internationally accepted diagnostic criteria system for dogs^20,21^whereas such systems are readily available for humans (DSM-5-TR)^22,41^).

Following the practices in human diagnostics as much as possible, we established criteria that integrate two results of the validated DAFRS questionnaire, the ADHD total score and the functional impairment measures, in order to differentiate dogs at-risk for ADHD. The predictive ability of the developed tool is reflected by the excellent AUC value of 0.861, which is comparable to AUC values of similar diagnostic questionnaire tools employed in human ADHD studies^46^. For instance, a systematic review of parent-rated ADHD questionnaires used as diagnostic tools reported AUC values ranging from 0.55 to 0.95^55^.

The sensitivity and specificity values that we achieved -good and excellent- are either consistent with or exceed those reported for human ADHD diagnostic tools^46,51,55,56^. Notably, these results are contingent upon the decisions made regarding the cutoff values. We prioritized specificity over sensitivity, which ​​favours less false-positive cases but increases the risk of missing actual, high risk ADHD cases. As our methodology can be regarded as an initial step of the diagnostic process, we aimed for a more stringent approach to ensure that the identified risk group accurately reflects a truly at-risk category. Importantly, this would help minimize unnecessary medication of dogs, as an increasing number of studies report that dogs with ADHD-like symptoms are often being medicated despite uncertain diagnostic criteria^18,20,21^. Although relevant behaviour test results demonstrated strong correlations with the DARFS data^57^ we cannot exclude that some dog owners lack adequate exposure to relevant situations, various breeds, or specific behavioural traits, which may result in inconsistencies in their responses to the questionnaire. Our choice of the cutoff point reflects the functional aspect of our approach, similar to the method used in some human diagnostics, when the aim is to avoid many false positive cases^51^.

Combining the ADHD score cutoff and the functional impairment criteria, about 4% of our sample population fell into the at-risk for ADHD category. While this value is consistent with the ADHD prevalence rates reported in adult human data^4^the prevalence in children and adolescents is typically higher, with recent estimates indicating global rates of approximately 5–9%^1,2,3^. The prevalence rate in the present sample can be affected by our prioritization of specificity over sensitivity, ensuring that only minimal false positives fall into the at-risk ADHD category. Moreover, this proportion is likely influenced by population-specific factors, including cultural differences and sample selection biases, similarly as in human research^6,58,59^. It is important to note that owners who fill out such questionnaires may not represent a random or unbiased sample^14^. Similar to the tendencies observed among parents of children with ADHD^27,60^ dog owners may underreport symptoms^14,61,62^ or manage the environment to mitigate perceived behavioural issues (e.g., reduced walking time due to impulsive actions^63^). Conversely, bias in the opposite direction is also possible, as owners who are more interested in behavioural issues and familiar with the topic, might be more likely to participate and to recognize or report symptoms. Therefore, it cannot be excluded that the actual prevalence of ADHD-like behaviours in dogs could be either under- or overrepresented in our data. Importantly, these at-risk dogs should undergo further examinations, such as relevant behavioural tests^57^ and expert consultations, which can help with differential diagnosis and refinement of classification.

It is also important to note that symptoms of hyperactivity and impulsivity may have been overrepresented among the at-risk for ADHD dogs. In human diagnostic practices, hyperactivity and impulsivity are often assessed concurrently due to their frequent co-occurrence, and this pattern may similarly apply to canines. Moreover, our prior research^14,57^ supports the assertion that symptoms of inattention are less observable and may be less disturbing to the environment compared to the more overt symptoms of hyperactivity and impulsivity. This might result in the underrepresentation of inattentive symptoms and associated impairments in our sample, aligning with findings from human studies^24,64^.

Notably, numerous dogs that only reached the cutoff score, or only fell into the functionally impaired criteria were not classified as being at-risk for ADHD. Our previous research indicated a significant correlation between functional impairment and ADHD total scores^14^ though the specific features contributing to this correlation were not examined. Despite scoring high on the ADHD total score (see Fig. 3), many dogs did not meet the criteria for functional impairment. Most of these dogs may be working or sporting dogs that had been selected for high activity and impulsivity, which, if properly treated, do not result in functional impairment^12,65^. On the other hand, some dogs fell into the functionally impaired category despite having lower ADHD total scores. This may have occurred when even a dog with non-extreme behaviour poses a problem for the owner.

The distribution of impairments in the present sample hints that some dogs exhibit impairments only in specific domains, such as inattention or hyperactivity, suggesting the possibility of ADHD subtypes in dogs, similar to those seen in humans^22,41^. Future studies should focus on less convenient, more targeted samples with more at-risk for ADHD dogs to better explore potential subtypes and to determine how well these align with human ADHD classifications.

While this study marks a significant step in identifying at-risk ADHD dogs using human analogue criteria, it also has limitations that need to be acknowledged. The cutoff points established in the current study may not be directly applicable to markedly different samples, such as smaller or less varied populations, where at-risk ADHD dogs may be underrepresented. Testing across diverse populations is essential to validate these findings, as cultural differences, sample size, sampling biases, and population-specific factors—similar to those in human ADHD research^6,58,59^ —can influence prevalence rates and diagnostic accuracy.

Further, data from multiple informants can provide a more comprehensive understanding of the individual’s behaviour by capturing diverse perspectives and contextual variations in symptom manifestations across different settings^24^. In contrast, not all dogs attend different settings such as dog schools or daycare, making it challenging to obtain cross-setting evaluation of behaviour. Moreover, this difference limits the ability to involve experts (trainers) in the evaluation process, which could help reduce owner bias, a factor that is also present in human diagnostics, where teacher assessments are an important complementary method to counterbalance parent bias^27^. Nonetheless, with the increasing trend of urban dogs participating in structured activities such as sports or obedience training, these differences may become less pronounced in the future.

To enhance the reliability of our methodology, it would be optimal to account for the level of training in the assessment process. This approach could, in the future, enable the development of different cutoff criteria tailored to dogs with varying levels of training, ensuring that the observed symptoms are not attributable to a lack of socialization and/or training, or insufficient exercise^12,21^.

It should be emphasized that the present sample was diverse in terms of both breed and age, and these factors may also significantly influence many aspects of canine behaviour and ADHD-like traits^12,21,65^. There is a possibility that owners may misinterpret the normal behaviours of juveniles or certain breeds as symptoms of hyperactivity and/or impulsivity, leading to potential misclassification. This is one reason why we decided to use a more cautious approach and apply a more rigorous criteria to identify dogs at-risk for ADHD. We acknowledge that formulating diagnostic criteria that can comprehensively consider all relevant factors (e.g., effects of breed, age, neutering status, environmental factors) is a complex task. Accordingly, this current study can be considered as the first step towards the application of more objective diagnostic criteria and measurement tools to identify dogs at-risk for ADHD.

Thus, it is clear that relying solely on the DAFRS questionnaire is not yet sufficient for diagnosing dogs suspected of ADHD. While the questionnaire provides a valuable foundation for screening, it should be supplemented with additional assessment methods. Observation of related behaviours^40,66^ and performing specific behavioural tests^57^ offer further insights into the symptoms and can help reduce the potential owner bias present in the questionnaire. Furthermore, expert consultations with a certified behaviour specialist veterinarian^18–21^ and, when feasible, involving the dog’s trainer in the evaluation^14,34^can provide a more comprehensive and accurate evaluation. Importantly, consultations should also inquire about the duration and onset of symptoms, as is standard in human ADHD diagnostics, where symptoms must persist continuously for at least six months. In the future, this multi-faceted approach would help differential diagnosis to rule out other behavioural problems and disorders that could coexist with ADHD-like behaviours and symptoms^11,17,67^ similarly to humans, where ADHD is highly comorbid^68–70^.

In conclusion, this study represents a significant advancement by establishing a human analogue diagnostic framework that transitions from treating ADHD traits as continuous variables to a categorical approach. Compared to the few published practices, we have developed a replicable method, offering greater reliability in identifying dogs at-risk for ADHD. Just as human diagnostic systems have evolved over decades with refined criteria, there is still room for enhancement in our canine diagnostic methodology, for example in establishing different thresholds for different samples.

Unlike previous studies that used ADHD-like traits for correlations with variables of interest of a given study, the current diagnostic criteria enable comparison between distinct groups (e.g., at-risk for ADHD vs. non-at risk for ADHD). This shift would not only mirror human research practices but also offers a more appropriate approach for studying dogs as models of human ADHD within a comparative framework.

## Electronic supplementary material

Below is the link to the electronic supplementary material.

Supplementary Material 1

## Data Availability

Data supporting the findings of this study are available within the paper and its Supplementary Information. The original data presented in the study are openly available in FigShare at https://doi.org/10.6084/m9.figshare.28207451.
