# Supplementary material for: Development of a human analogue ADHD diagnostic system for family dogs
Source: Sci Rep. 2025 Jul 16;15:25671. doi: 10.1038/s41598-025-09988-8 (PMC12263975; doi:10.1038/s41598-025-09988-8)
Supplement: Supplementary file 1 — Supplementary Material 1 [file 41598_2025_9988_MOESM1_ESM.docx]

**Supplementary material to:**

**Development of a human analogue ADHD diagnostic system for family dogs**

**Barbara Csibra ^1^ *, Nóra Bunford ^2^ and Márta Gácsi ^1,3^**

^1^ Eötvös Loránd University, Institute of Biology, Department of Ethology, Pázmány Péter sétány 1/C, Budapest, 1117 Hungary;

^2^ Clinical and Developmental Neuropsychology Research Group, Research Centre for Natural Sciences, Institute of Cognitive Neuroscience and Psychology, Magyar tudósok körútja 2, Budapest, 1117 Hungary;

^3^ HUN-REN-ELTE Comparative Ethology Research Group, Pázmány Péter sétány 1/C, Budapest, 1117, Hungary;

* Correspondence: csibrabarbara@gmail.com

Appendix A: Dog ADHD and Functionality Rating Scale (DAFRS), owner-report form ^1^.

Space was provided for answers where necessary. Asterisk-marked questions are not mandatory to be answered. Q: questions; IA = inattention; H = hyperactivity; I = Impulsivity. Functionality item categorization by the related impairments: F-IA = functionality-inattention; F-H: functionality-hyperactivity; F-I = functionality-impulsivity; F-A = functionality-aggression; F-V = functionality-vocalization. Given that aggression-related functional impairments in dogs cannot be exclusively attributed to ADHD traits and may have been influenced by other factors (e.g., gender, neutering status, education, etc.), we consequently omitted the aggression-related impairment area from the analyses pertaining to functionality. We also omitted the two vocalization related items from the functionality evaluation. Similarly to aggression, vocalization in dogs cannot be exclusively attributed to ADHD traits and may have been influenced by factors such as breed, training, or environmental stressors.

| Description of the questionnaire for owners | | | |
| --- | --- | --- | --- |
| Dear Owner! The main goal of the present questionnaire is to measure the dogs' level of attention, activity, and impulsivity. Completing the questionnaire takes approximately 15 minutes, and we hope you will find it interesting! There are no right or wrong answers, but please, answer all questions to the best of your knowledge! Our goal is to get the most accurate picture of your dog. Please complete the questionnaire for one dog at a time. If you want to complete it for additional dogs, please re-open the questionnaire and submit a new response. Data are stored for the sole purpose of communication regarding participation in research and are not communicated to any third parties. Thank you for helping us! | | | |
| Consent statement and privacy policy | | | |
| After data are processed and analysed, results will be published in the form of scientific presentations, papers, or educational materials. During data processing, data are stored at the Department of Ethology at Eötvös Loránd University. Research participants are informed about results upon reasonable request. Participation in this research is voluntary and anonymous, and the data obtained is used for scientific purposes only. In compliance with relevant data protection laws, personally identifying data are treated confidentially and stored separately from the rest of research data. Data sets shared with other researchers or made publicly available do not contain identifying information. Research data are further handled as part of a large database. Should you have any questions or comments regarding this research, please, contact our research team: Barbara Csibra, PhD student, ELTE Department of Ethology, email: csibrabarbara@gmail.com | | | |
| Consent Statement: I have read the consent and privacy policy information and am aware of the goals of- and processes involved in the current research. By participating in this research, I consent to my anonymized data being shared and treated as part of a larger database. I am aware that I may discontinue participating in this research at any time. | | | Yes/No (The questionnaire is terminated if you answer "No.") |
| GENERAL QUESTIONS | | | |
| Q | Subscale/Facet | Item description | Scale and scoring |
| 1 | - | Owner's name (or unique identifier) (Data are stored for the sole purpose of communication regarding participation in research and are not communicated to any third parties.) | Text |
| 2 | - | Email address (It is used only for the communication necessary for the tests and is not passed on to third parties.) | Text |
| 3 | - | The dog's name | Text |
| 4 | - | The dog's date of birth in YYYY.MM.DD format. If you DO NOT KNOW your dog's exact birth date, please, enter the following numbers: "1212.12.12." and go to the next question where you can enter the estimated age of the dog. | Text |
| 5 | - | Your dog's estimated date of birth. If you don't know exactly when your dog was born, please estimate its age. * | Text |
| 6 | - | The dog's breed | Text |
| 7 | - | If you selected "other breed", please describe the breed here: * | Text |
| 8 | - | The dog's sex and neutering status | male, female, neutered male, neutered female |
| 9 | - | What kind of training has your dog received? | (0) None or I trained him/her at home – (1) Basic obedience (dog-school/trainer) – (2) Higher obedience (dog-school) |
| QUESTIONS ON ATTENTION, ACTIVITY, IMPULSIVITY | | | |
|  |  | How typical are the following statements of your dog? |  |
| 1 | H | Fidgets, bustles. | (0) Never – (1) Rarely – (2) Often – (3) Very often |
| 3 | I | Becomes very excited when facing a new, mildly stressful situation (e.g., facing new situation/place or meeting new people/dogs). |  |
| 4 | I | Once your dog "gets going", it is difficult to hold him/her back or stop. |  |
| 7 | I | Has no self-control. |  |
| 12 | IA | Has difficulties concentrating. |  |
| 13 | I | Is excessive, unrestrained, rampant. |  |
| 14 | H | Is rarely calm, even in familiar places, calm situations (e.g., at home). |  |
| 17 | I | Is difficult to calm down. |  |
| 20 | H | Active even after fatiguing or hard exercise/ work. |  |
| 24 | IA | Has difficulties with learning, because he/she does not pay attention. |  |
| 25 | IA | When your dog is asked to perform a task, he/she is reluctant to comply or withdraws from the situation. |  |
| 26 | IA | Performs poorly on tasks that require a lot of thinking. |  |
| 27 | IA | Has difficulties (makes many mistakes) with completing complicated tasks, even if he/she is familiar with the task and has frequently practiced he/she before. |  |
| 29 | IA | Quickly loses intertest. |  |
| 33 | H | Would always play and run. |  |
| 38 | I | Is difficult to control and handle. |  |
| 42 | I | Reacts rashly to new stimuli, without considering the consequences. |  |
| QUESTIONS ON FUNCTIONALITY | | | |
|  |  | In the case of your dog, to what extent are the following problems (if they occur) attributable to the dog’s inattention? (For example, in the case of the first question, to what extent is your dog's inattention responsible for your concern about releasing it?) |  |
| 1 | F-IA | Cannot be released in an unfamiliar place | (0) There is no such problem –  (0) It is a problem, but not a result of inattention – (1) It is a problem, and, to some extent, is a result of inattention – (2) It is a problem, and, more or less, is a result of inattention – (3) It is a problem, and is largely a result of inattention |
| 2 | F-IA | Is unable to engage in activities with other dogs in a conflict-free manner, e.g., play with them, get to know them |  |
| 3 | F-IA | It learns with difficulty and slowly, as indicated, for example, by it lagging behind in dog school |  |
| 4 | F-IA | Other dog owners don't like walking with us |  |
| 5 | F-IA | Annoys, bothers guests and relatives, e.g., by jumping on them, nipping them |  |
| 6 | F-IA | I am concerned to release it in the presence of a toddler (I'm afraid it will knock the toddler over) |  |
| 7 | F-IA | It disrupts mobility in the home/garden |  |
|  |  | In the case of your dog, to what extent are the following problems (if they occur) attributable to the dog’s impulsivity? (For example, in the case of the first question, to what extent is your dog's impulsivity responsible for your concern about releasing it?) |  |
| 8 | F-I | Cannot be released in an unfamiliar place | (0) There is no such problem –  (0) It is a problem, but not a result of impulsivity – (1) It is a problem, and, to some extent, is a result of impulsivity – (2) It is a problem, and, more or less, is a result of impulsivity – (3) It is a problem, and is largely a result of impulsivity |
| 9 | F-I | Is unable to engage in activities with other dogs in a conflict-free manner, e.g., play with them, get to know them |  |
| 10 | F-I | It learns with difficulty and slowly, as indicated, for example, by it lagging behind in dog school |  |
| 11 | F-I | Other dog owners don't like walking with us |  |
| 12 | F-I | Annoys, bothers guests and relatives, e.g., by jumping on them, nipping them |  |
| 13 | F-I | I am concerned to release it in the presence of a toddler (I'm afraid it will knock the toddler over) |  |
| 14 | F-I | It disrupts mobility in the home/garden |  |
|  |  | In the case of your dog, to what extent are the following problems (if they occur) attributable to the dog’s excessive activity? (For example, in the case of the first question, to what extent is your dog's excessive activity responsible for your concern about releasing it?) |  |
| 15 | F-H | Cannot be released in an unfamiliar place | (0) There is no such problem –  (0) It is a problem, but not a result of excessive activity – (1) It is a problem, and, to some extent, is a result of excessive activity – (2) It is a problem, and, more or less, is a result of excessive activity – (3) It is a problem, and is largely a result of excessive activity |
| 16 | F-H | Is unable to engage in activities with other dogs in a conflict-free manner, e.g., play with them, get to know them |  |
| 17 | F-H | It learns with difficulty and slowly, as indicated, for example, by it lagging behind in dog school |  |
| 18 | F-H | Other dog owners don't like walking with us |  |
| 19 | F-H | Annoys, bothers guests and relatives, e.g., by jumping on them, nipping them |  |
| 20 | F-H | I am concerned to release it in the presence of a toddler (I'm afraid it will knock the toddler over) |  |
| 21 | F-H | It disrupts mobility in the home/garden |  |
|  |  | In the case of your dog, to what extent are the following problems (if they occur) attributable to the dog’s aggression? (For example, in the case of the first question, to what extent is your dog's aggression responsible for your concern about releasing it?) |  |
| 22 | F-A | Cannot be released in an unfamiliar place | (0) There is no such problem –  (0) It is a problem, but not a result of aggression – (1) It is a problem, and, to some extent, is a result of aggression – (2) It is a problem, and, more or less, is a result of aggression – (3) It is a problem, and is largely a result of aggression |
| 23 | F-A | Is unable to engage in activities with other dogs in a conflict-free manner, e.g., play with them, get to know them |  |
| 24 | F-A | It learns with difficulty and slowly, as indicated, for example, by it lagging behind in dog school |  |
| 25 | F-A | Other dog owners don't like walking with us |  |
| 26 | F-A | Annoys, bothers guests and relatives, e.g., by jumping on them, nipping them |  |
| 27 | F-A | I am concerned to release it in the presence of a toddler (I'm afraid it will knock the toddler over) |  |
| 28 | F-A | It disrupts mobility in the home/garden |  |
|  |  | How typical are the following statements of your dog? |  |
| 29 | F-V | Cannot be quiet, whines or barks a lot even when there is nothing special to evoke this. | (0) Never – (1) Rarely – (2) Often – (3) Very often |
| 30 | F-V | If your dog starts to bark or whine, it is difficult to silence him/her. |  |

Appendix B: Weighting procedure

Given the different number of items per ADHD subscale (inattention: 6 items; hyperactivity: 4 items; impulsivity: 7 items), a weighting procedure was required to ensure that each subscale contributes equally to the ADHD total score. To equalize the maximum possible subscale scores for all ADHD subscales, the highest maximum item number, which is 7 from the impulsivity subscale, was used as the reference. The weighting factors were calculated by dividing this maximum item count by the maximum item counts of the other subscales. This procedure was not needed to be performed on the functionality-related items, as all functionality impairment areas were represented with 7 items. The calculated weighting factors are presented in Table 1.

**Table 1.** Weighting factors for the different DAFRS subscales. Note: The maximum possible subscale score is obtained by multiplying the maximum item count by 3, because we used a 4-point rating scale in the questionnaire, with a maximum score of 3 available for each item, depending on how frequently the behaviour described in the question occurs (0 = never; 1 = sometimes; 2 = often; 3 = very often).

| Subscale | Maximum item count | Maximum subscale score | Weighting Factor |
| --- | --- | --- | --- |
| Inattention | 6 | 3 x 6 = 18 | 7/6 = 1.167 |
| Hyperactivity | 4 | 3 x 4 = 12 | 7/4 = 1.75 |
| Impulsivity | 7 | 3 x 7 = 21 | 7/7 = 1 |

These weighting factors were then applied to each subscale scores. Specifically, each subscale's score was multiplied by its respective weighting factor to adjust its contribution to the weighted ADHD total score. The weighted ADHD total score was obtained by summing the weighted subscale scores of the three subscales.

As an example, if a respondent received a score of 4 on the inattention subscale, 3 on the hyperactivity subscale, and 5 on the impulsivity subscale, the weighted subscale scores would be 4.67 for inattention (4 * 1.167), 5.25 for hyperactivity (3 * 1.75), and 5 for impulsivity (5 * 1). The weighted total ADHD score is the sum of these weighted subscale scores, resulting in approximately 14.92.

Following the application of these weighting factors, the maximum possible item count for each ADHD subscale is standardized to 7. Consequently, the maximum subscale score per scale is standardized to 21, thus the maximum weighted ADHD total score is 63 after the procedure. For the ROC analysis, we rounded the weighted ADHD total scores to whole numbers and used them in the analysis accordingly.

Appendix C: Receiver Operating Characteristic (ROC) analysis

Receiver Operating Characteristic (ROC) analysis is a commonly applied statistical method to assess the accuracy of a diagnostic test in distinguishing between disorder presence and disorder absence ^2,3^. The method evaluates the test’s sensitivity (true positive rate) and specificity (true negative rate) across different cutoff values, helping researchers to determine an optimal threshold that balances both measures ^2^. The discriminatory ability of the test can be inspected via the ROC curve, which shows the true positive rate against the false positive rate at different cutoff values (Habibzadeh et al. 2016). The area under the ROC curve (AUC) shows the overall accuracy of the measure, with a higher AUC indicating a stronger discriminatory capability. The AUC ranges from 0.5 (45° diagonal line, uninformative test) to 1.0 (perfect test) on the ROC curve ^2,4^, with values interpreted as follows: 0.51–0.69 signifies a poor test, 0.7–0.79 an acceptable or fair test, 0.8–0.89 an excellent test, and 0.9 or above is considered outstanding ^5,6^. Each point on a ROC curve represents a specific cutoff value and is linked to both sensitivity and specificity of the test ^2^. Determining the optimal cutoff point requires a compromise between sensitivity and specificity. For instance, sensitivity might be prioritized over specificity when dealing with highly infectious diseases or conditions with severe complications. Conversely, specificity may be favored in situations where further diagnostic testing is either risky or expensive. If no particular preference exists between sensitivity and specificity, the typical approach is to maximize both indices. Sensitivity and specificity values above 80% were considered as excellent outcomes, between 70% and 79% as good, between 60% and 69% fair, and below 60% poor outcomes for a diagnostic test ^7,8^.

Appendix D: Sensitivity and specificity values for various weighted ADHD total score thresholds. The final cutoff score along with the corresponding sensitivity and specificity values are indicated in bold in the table. Note: For the ROC analysis, we rounded the weighted ADHD total scores to whole numbers and used them in the analysis accordingly. The smallest cutoff value is the minimum observed test value minus 1, and the largest cutoff value is the maximum observed test value plus 1. All the other cutoff values are the averages of two consecutive ordered observed test values.

| **Weighted ADHD total score** | **Sensitivity** | **Sensitivity (%)** | **1 - Specificity** | **Specificity (%)** |
| --- | --- | --- | --- | --- |
| -1,00 | 1,00 | 100,00 | 1,00 | 0,00 |
| 0,50 | 1,00 | 100,00 | 0,99 | 0,80 |
| 1,50 | 1,00 | 100,00 | 0,99 | 1,37 |
| 2,50 | 1,00 | 100,00 | 0,98 | 2,39 |
| 3,50 | 1,00 | 100,00 | 0,96 | 3,87 |
| 4,50 | 1,00 | 100,00 | 0,94 | 6,32 |
| 5,50 | 1,00 | 100,00 | 0,90 | 9,74 |
| 6,50 | 1,00 | 100,00 | 0,87 | 13,44 |
| 7,50 | 1,00 | 100,00 | 0,83 | 17,37 |
| 8,50 | 1,00 | 100,00 | 0,78 | 22,32 |
| 9,50 | 1,00 | 100,00 | 0,74 | 25,63 |
| 10,50 | 1,00 | 100,00 | 0,71 | 29,50 |
| 11,50 | 1,00 | 100,00 | 0,67 | 32,74 |
| 12,50 | 1,00 | 100,00 | 0,64 | 36,22 |
| 13,50 | 0,98 | 98,28 | 0,59 | 40,72 |
| 14,50 | 0,97 | 97,41 | 0,55 | 45,22 |
| 15,50 | 0,97 | 96,55 | 0,51 | 48,58 |
| 16,50 | 0,94 | 93,97 | 0,47 | 53,47 |
| 17,50 | 0,92 | 92,24 | 0,42 | 57,69 |
| 18,50 | 0,92 | 92,24 | 0,39 | 60,99 |
| 19,50 | 0,90 | 89,66 | 0,35 | 64,75 |
| 20,50 | 0,85 | 85,34 | 0,32 | 68,39 |
| 21,50 | 0,84 | 84,48 | 0,28 | 71,53 |
| 22,50 | 0,80 | 80,17 | 0,25 | 74,89 |
| 23,50 | 0,78 | 77,59 | 0,23 | 77,45 |
| 24,50 | 0,73 | 73,28 | 0,20 | 80,24 |
| **25,50** | **0,71** | **70,69** | **0,18** | **82,46** |
| 26,50 | 0,66 | 65,52 | 0,15 | 84,79 |
| 27,50 | 0,63 | 62,93 | 0,14 | 86,28 |
| 28,50 | 0,58 | 57,76 | 0,13 | 87,47 |
| 29,50 | 0,57 | 56,90 | 0,11 | 88,84 |
| 30,50 | 0,54 | 54,31 | 0,09 | 90,66 |
| 31,50 | 0,51 | 50,86 | 0,08 | 92,08 |
| 32,50 | 0,46 | 45,69 | 0,07 | 93,05 |
| 33,50 | 0,41 | 40,52 | 0,06 | 94,08 |
| 34,50 | 0,41 | 40,52 | 0,05 | 95,10 |
| 35,50 | 0,35 | 35,34 | 0,04 | 95,96 |
| 36,50 | 0,34 | 33,62 | 0,03 | 96,64 |
| 37,50 | 0,32 | 31,90 | 0,03 | 97,15 |
| 38,50 | 0,27 | 26,72 | 0,03 | 97,44 |
| 39,50 | 0,25 | 25,00 | 0,02 | 97,84 |
| 40,50 | 0,23 | 23,28 | 0,02 | 98,35 |
| 41,50 | 0,20 | 19,83 | 0,01 | 98,52 |
| 42,50 | 0,16 | 16,38 | 0,01 | 98,80 |
| 43,50 | 0,13 | 12,93 | 0,01 | 99,03 |
| 44,50 | 0,11 | 11,21 | 0,01 | 99,26 |
| 45,50 | 0,11 | 11,21 | 0,01 | 99,37 |
| 46,50 | 0,09 | 9,48 | 0,00 | 99,54 |
| 47,50 | 0,09 | 8,62 | 0,00 | 99,77 |
| 48,50 | 0,08 | 7,76 | 0,00 | 99,89 |
| 49,50 | 0,08 | 7,76 | 0,00 | 99,94 |
| 50,50 | 0,07 | 6,90 | 0,00 | 99,94 |
| 51,50 | 0,06 | 6,03 | 0,00 | 99,94 |
| 52,50 | 0,04 | 4,31 | 0,00 | 100,00 |
| 54,00 | 0,03 | 2,59 | 0,00 | 100,00 |
| 56,00 | 0,02 | 1,72 | 0,00 | 100,00 |
| 59,00 | 0,01 | 0,86 | 0,00 | 100,00 |
| 62,00 | 0,00 | 0,00 | 0,00 | 100,00 |

Appendix E: Detailed information on sample demographics of the at-risk ADHD dogs. The identified at-risk ADHD group consisted of 79 dogs, including 29 mixed-breed dogs and 50 purebred dogs from 28 distinct breeds. The group comprised 47 males and 32 females, of which 59 were neutered and 20 intact, the age of the dogs ranged from 10 to 180 months (*M*age = 43.59 months, *SD* = 31.37).

| **Breed** | **Age (months)** | **Sex** | **Neutering status** |
| --- | --- | --- | --- |
| Mixed Breed | 10 | Female | Intact |
| Pumi | 10 | Male | Intact |
| Mixed Breed | 12 | Male | Neutered |
| Pumi | 12 | Male | Neutered |
| Mixed Breed | 12 | Male | Neutered |
| Mudi | 13 | Male | Intact |
| Mixed Breed | 13 | Male | Neutered |
| Mixed Breed | 15 | Male | Neutered |
| Mixed Breed | 16 | Male | Neutered |
| English Cocker Spaniel | 17 | Male | Intact |
| Mixed Breed | 18 | Male | Neutered |
| Mixed Breed | 19 | Male | Neutered |
| Irish Setter | 20 | Female | Intact |
| Kuvasz | 20 | Female | Intact |
| English Cocker Spaniel | 21 | Male | Intact |
| Mixed Breed | 21 | Male | Intact |
| Welsh Corgi - Pembroke | 21 | Male | Intact |
| Bull Terrier | 22 | Female | Neutered |
| Mixed Breed | 22 | Male | Intact |
| Miniature Pinscher | 22 | Male | Neutered |
| Mixed Breed | 23 | Female | Intact |
| Mixed Breed | 24 | Female | Neutered |
| Gordon Setter | 24 | Female | Neutered |
| Mixed Breed | 24 | Male | Neutered |
| English Cocker Spaniel | 24 | Male | Neutered |
| Border collie | 24 | Male | Neutered |
| Mudi | 25 | Male | Intact |
| American Staffordshire Terrier | 26 | Male | Neutered |
| Mixed Breed | 27 | Female | Neutered |
| Vizsla | 27 | Male | Neutered |
| Mixed Breed | 28 | Female | Neutered |
| Mixed Breed | 28 | Male | Neutered |
| Standard Poodle | 29 | Female | Neutered |
| Mudi | 30 | Male | Intact |
| Mixed Breed | 31 | Female | Neutered |
| Giant Schnauzer | 32 | Male | Intact |
| Mixed Breed | 32 | Male | Neutered |
| Shiba Inu | 33 | Female | Intact |
| Irish Setter | 34 | Female | Intact |
| Labrador Retriever | 34 | Male | Neutered |
| American Staffordshire Terrier | 36 | Female | Neutered |
| Fox Terrier | 37 | Female | Neutered |
| French Bulldog | 37 | Female | Neutered |
| Staffordshire Bull Terrier | 39 | Female | Neutered |
| Border collie | 39 | Female | Neutered |
| Fox Terrier | 39 | Male | Neutered |
| American Staffordshire Terrier | 40 | Male | Neutered |
| Old English Sheepdog (Bobtail) | 42 | Male | Neutered |
| Caucasian Shepherd Dog | 43 | Male | Neutered |
| Mixed Breed | 44 | Female | Neutered |
| Mixed Breed | 46 | Female | Neutered |
| German Shepherd Dog | 48 | Male | Intact |
| Mixed Breed | 48 | Male | Neutered |
| Mixed Breed | 48 | Male | Neutered |
| Chow Chow | 49 | Male | Neutered |
| Mixed Breed | 49 | Male | Neutered |
| Vizsla | 53 | Male | Intact |
| Mixed Breed | 55 | Male | Neutered |
| Pug | 56 | Female | Neutered |
| Border collie | 59 | Female | Neutered |
| German Shepherd Dog | 59 | Female | Neutered |
| Fox Terrier | 60 | Male | Neutered |
| Mixed Breed | 60 | Male | Neutered |
| Mudi | 60 | Male | Neutered |
| Parson Russell Terrier | 63 | Female | Neutered |
| Mixed Breed | 66 | Male | Neutered |
| Bolognese | 67 | Male | Intact |
| French Bulldog | 69 | Female | Intact |
| Mudi | 75 | Female | Neutered |
| Mudi | 76 | Female | Neutered |
| Chinese Crested Dog | 77 | Male | Neutered |
| Mixed Breed | 77 | Male | Neutered |
| Mixed Breed | 79 | Male | Neutered |
| English Cocker Spaniel | 89 | Female | Neutered |
| Miniature Pinscher | 96 | Female | Neutered |
| German Shepherd Dog | 97 | Female | Neutered |
| Mixed Breed | 140 | Male | Neutered |
| Bull Terrier | 152 | Female | Neutered |
| Maltese | 180 | Female | Neutered |

**References**

1. Csibra, B., Bunford, N. & Gácsi, M. Development of a human-analogue, 3-symptom domain Dog ADHD and Functionality Rating Scale (DAFRS). *Sci Rep* **14**, 1808 (2024).

2. Habibzadeh, F., Habibzadeh, P. & Yadollahie, M. On determining the most appropriate test cut-off value: the case of tests with continuous results. *Biochem Med (Zagreb)* **26**, 297–307 (2016).

3. Kamarudin, A. N., Cox, T. & Kolamunnage-Dona, R. Time-dependent ROC curve analysis in medical research: current methods and applications. *BMC Medical Research Methodology* **17**, 53 (2017).

4. Christiansen, H. *et al.* German validation of the Conners Adult ADHD Rating Scales (CAARS) II: Reliability, validity, diagnostic sensitivity and specificity. *Eur. psychiatr.* **27**, 321–328 (2012).

5. Carter, J. V., Pan, J., Rai, S. N. & Galandiuk, S. ROC-ing along: Evaluation and interpretation of receiver operating characteristic curves. *Surgery* **159**, 1638–1645 (2016).

6. Mandrekar, J. N. Receiver Operating Characteristic Curve in Diagnostic Test Assessment. *Journal of Thoracic Oncology* **5**, 1315–1316 (2010).

7. Leonardi Dutra, K. *et al.* Diagnostic Accuracy of Cone-beam Computed Tomography and Conventional Radiography on Apical Periodontitis: A Systematic Review and Meta-analysis. *Journal of Endodontics* **42**, 356–364 (2016).

8. Major, M. P. *et al.* The accuracy of diagnostic tests for adenoid hypertrophy: A systematic review. *The Journal of the American Dental Association* **145**, 247–254 (2014).
